# A Comparative Pharmacodynamic Study of Ticagrelor versus Clopidogrel and Ticagrelor in Patients Undergoing Primary Percutaneous Coronary Intervention: The CAPITAL RELOAD Study

**DOI:** 10.1371/journal.pone.0092078

**Published:** 2014-03-20

**Authors:** Benjamin Hibbert, Ronnen Maze, Ali Pourdjabbar, Trevor Simard, F. Daniel Ramirez, Rohit Moudgil, Melissa Blondeau, Marino Labinaz, Alexander Dick, Christopher Glover, Michael Froeschl, Jean-François Marquis, Derek Y. F. So, Michel R. Le May

**Affiliations:** Division of Cardiology, University of Ottawa Heart Institute, Ottawa, Ontario, Canada; King’s College London School of Medicine, United Kingdom

## Abstract

**Background:**

In patients undergoing primary percutaneous coronary intervention (PPCI) ticagrelor is superior to clopidogrel in reducing cardiovascular events. This study sought to evaluate the effect of clopidogrel pretreatment on the pharmacodynamics of ticagrelor in patients undergoing PPCI.

**Methods:**

We measured platelet reactivity using the VerifyNow P2Y12 assay at baseline, 1, 2, 4, 6, 12, 24, and 48 hours following ticagrelor bolus in patients previously loaded with clopidogrel (C+T) and in thienopyridine-naive patients (T) referred to our centre for PPCI.

**Results:**

In total, 52 consecutive eligible patients with ST-elevation myocardial infarction (STEMI) were enrolled (27 C+T and 25 T). Baseline characteristics and mean baseline platelet reactivity units (PRUs) were similar between the groups. The primary endpoint, the proportion of patients achieving a PRU<208 at 2 hours, was more frequently achieved in the C+T group compared to T treatment (76.0% vs 44.4%, p = 0.026). Notably, C+T therapy resulted in fewer patients with high platelet reactivity at 1 hour (56.0% vs. 14.8%), 4 hours (100.0% vs. 61.5%) and 6 hours (100.0% vs. 64%, p<0.01 for all comparisons). Furthermore, C+T therapy was associated with lower PRU values from 2 to 48 hours.

**Conclusions:**

In patients referred for PPCI, ticagrelor bolus following clopidogrel resulted in more rapid and profound platelet inhibition, demonstrating a positive pharmacodynamic interaction. Further study is needed to determine if this pharmacodynamic effect translates into reduced clinical events.

## Introduction

Patients undergoing primary percutaneous coronary intervention (PPCI) for ST-elevation myocardial infarction (STEMI) are routinely treated with a P2Y12 inhibitor in addition to aspirin. Recently, ticagrelor was shown to be superior to clopidogrel in acute coronary syndrome (ACS) patients in the PLATelet inhibition and patient Outcomes (PLATO) trial [1]. Notably, in the cohort of STEMI patients, ticagrelor use was associated with a reduction in myocardial infarction (MI), stent thrombosis (ST), and overall mortality [2]. Accordingly, current guidelines recommend that ticagrelor (or prasugrel) be administered to patients with STEMI undergoing PPCI [3]–[5].

Rapid inhibition of platelet aggregation is paramount in patients undergoing PCI as delayed onset of drug activity or high on treatment platelet reactivity (HPR) is associated with increased risk of cardiovascular events, including ST [6]. Unlike clopidogrel and prasugrel which require biotransformation into active metabolites, ticagrelor is orally active in its parent form and in healthy volunteers results in inhibition of platelet aggregation as early as 2 hours following administration [7]. Most recently, pharmacodynamics reported in 25 patients undergoing PPCI and treated with ticagrelor suggested that onset of antiplatelet activity may be delayed in patients with STEMI [8]. These findings were subsequently confirmed in a second pharmacodynamic study [9]. Thus, identifying factors which improve the pharmacodynamic profile of P2Y12 inhibitors may enable more efficacious antiplatelet regimens.

In the PLATO trial, over 40% of patients received pre-randomization clopidogrel at doses ranging from 75–600 mg [1]. In the STEMI cohort of this study, a trend towards benefit was observed with higher doses of pre-randomization clopidogrel [2]. This is particularly noteworthy given that data from both the Response to Ticagrelor in Clopidogrel Nonresponders and Responders and Effect of Switching Therapies Study (RESPOND) and the Dose confirmation Study assessing anti-Platelet Effects of AZD6140 vs. clopidogrel in non-ST-segment Elevation myocardial infarction (DISPERSE-2) trial suggested that clopidogrel administration prior to ticagrelor may result in a positive pharmacodynamic interaction [10], [11]. The biological plausibility of this interaction is supported by ticagrelor binding the P2Y12 receptor at a site distinct from the ADP binding site targeted by clopidogrel [12]. Thus, we set out to determine the pharmacodynamic profile of ticagrelor in STEMI and to investigate the effect of reloading patients with ticagrelor who have already received a clopidogrel loading dose.

## Methods

### Study Design and Patients

The University of Ottawa Heart Institute regional STEMI program serves a population of approximately 1.3 million residents in eastern Ontario, receiving patients from 17 referral hospitals as well as direct transport by paramedics from the field [13]–[15]. The current study was a prospective observational cohort study performed on consecutive eligible patients referred for PPCI between June 2012 and November 2012. Inclusion required ischemic chest discomfort lasting greater than 30 minutes and less than 12 hours with ST segment elevation of greater than 1 mm in two or more contiguous electrocardiographic leads on a standard 12-lead EKG. Exclusion criteria included age <18 years, active bleeding, inadequate vascular access, use of an oral anticoagulant, known coagulopathy, allergy to antiplatelet therapy, severe renal impairment, severe contrast allergy, previous bypass surgery, PCI in the preceeding 12 months, current treatment with a thienopyridine, and administration of ticagrelor prior to obtaining a baseline blood sample.

All patients undergoing PPCI at our center receive a ticagrelor 180 mg loading dose irrespective of previous administration of clopidogrel by a referring center. During the enrollement period of this study, referral centers bolused patients with 600 mg of clopidogrel prior to transfer. Thus, we elected to perform an open label non-randomized prospective study to understand the effect of clopidogrel co-therapy in patients receiving bolus ticagrelor for PPCI. The groups consisted of patients who received ticagrelor 180 mg bolus alone (T) or patients who had received a bolus of clopidogrel 600 mg, at the discretion of the point of medical contact physician, and were then reloaded with ticagrelor prior to PPCI (C+T). All patients subsequently received ticagrelor 90 mg twice daily. Patients were consented on arrival to the PCI center. Adjuvant pharmacotherapy included aspirin 160 mg to chew followed by 81 mg daily, an unfractionated heparin bolus of 60 units/kg to a maximum of 4,000 units followed by bivalirudin administered at the time of PCI as a bolus of 0.75 mg/kg followed by an infusion of 1.75 mg/kg/hr during the procedure and at 0.25 mg/kg/hr afterward for a total of 2 hours.

### Outcome Measures

Inhibition of platelet reactivity was assessed by the VerifyNow P2Y12 assay and reported as platelet reactivity units (PRU). Blood samples were collected in 2.7 mL citrate (3.2%) tubes by venipuncture and analyzed as per manufacturer’s instructions. Baseline blood samples were defined as time point zero and were drawn just prior to ticagrelor bolus, which was administered during transfer to the catheterization laboratory. Samples were subsequently assessed at 1, 2, 4, 6, 12, 24, and 48 hours. The primary outcome was the proportion of patients achieving PRU<208 at 2 hours. Secondary outcomes included comparison of absolute PRU values assessed as a continuous variable in addition to the proportion of patients achieving a PRU<208 at the remaining time points. Baseline demographic data was collected prospectively on all patients as well as assessment of hemoglobin concentration, platelet count, and mean platelet volume at baseline and at 48 hours. Serial creatinine kinase (CK) serum levels were collected and infarct size estimated by peak CK values [16].

### Statistical Analysis

All continuous variables were described as mean (± standard deviation) or median (and interquartile range) as appropriate and categorical variables as number (%). Categorical variables were compared by Fisher exact test or Chi-square test and continuous variables by student t-test or Mann-Whitney rank sum test, as appropriate. Correlation between PRU values and MI size was assessed using a Pearson correlation coefficient. To calculate sample size, we estimated that 20% of patients in the T arm would achieve PRUs<208 at 2 hours compared to 60% of patients in the C+T arm [17]. Thus, using α = 0.05 and a power of 80%, we calculated a necessary sample size of 23 patients per group. We increased this to a minimum of 25 patients per group assuming a 10% drop out rate due to death, ST, or assay malfunction. All analyses were performed using Sigmastat version 3.5. Statistical significance was defined as p<0.05.

### Ethics Statement

This study was reviewed and approved by the University of Ottawa Heart Institute human research ethics board and written informed consent was obtained. The study protocol conforms to the ethical guidelines of the 1975 Declaration of Helsinki.

## Results

### Population and Baseline Characteristics

**Figure 1:**
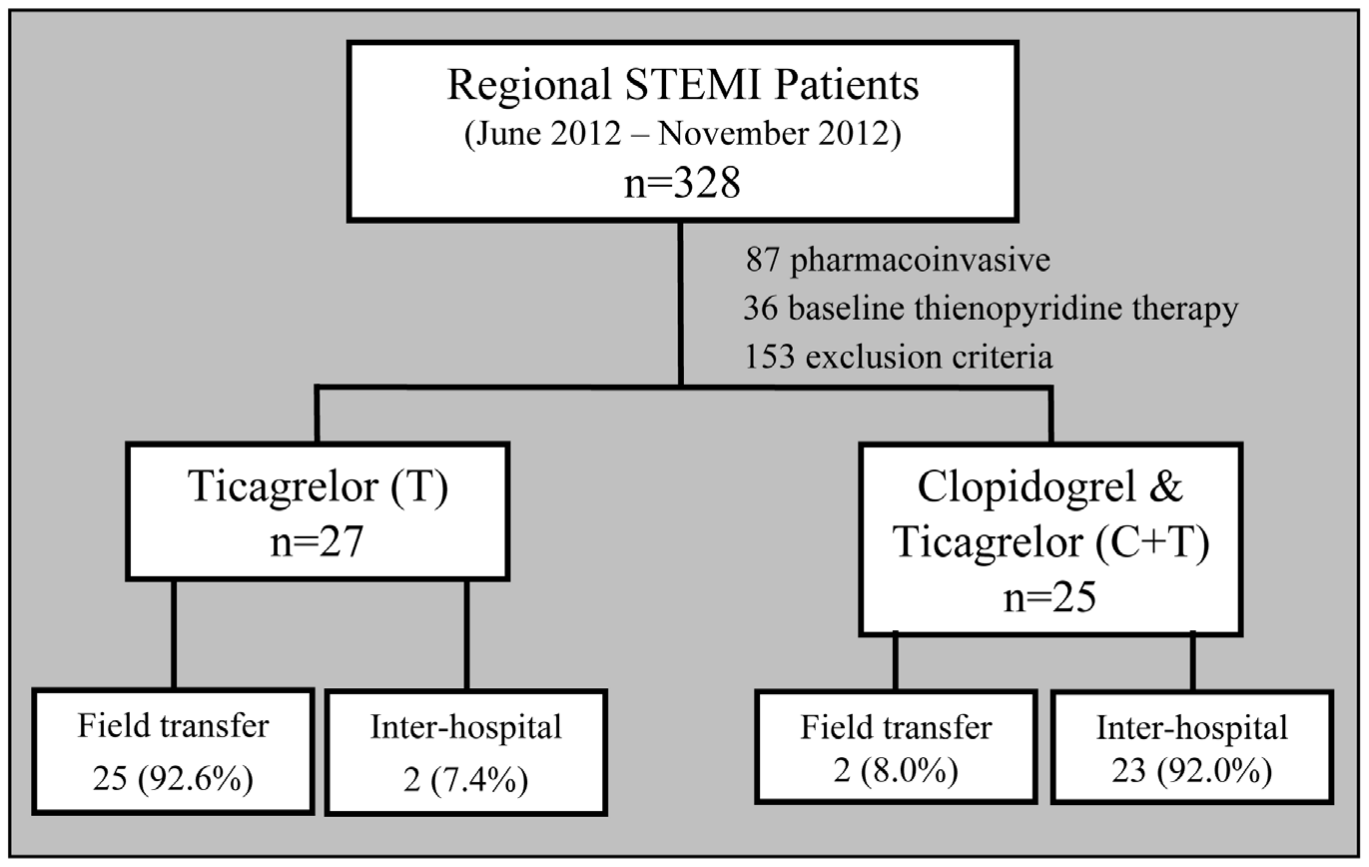
Selection of study population. Patients referred to the PCI center for primary PCI by a regionalized STEMI program. Consecutive eligible patients were recruited for serial platelet reactivity testing using the VerifyNow P2Y12 assay.

From June 2012 to November 2012, 328 patients were indexed in our regional STEMI program of which 52 eligible patients were enrolled and underwent baseline and serial PRU testing with the VerifyNow P2Y12 assay (**Figure 1**). Of patients excluded, 87 patients were treated with a pharmacoinvasive strategy, 36 patients were on chronic clopidogrel therapy, and 153 patients had other exclusion criteria, including administration of ticagrelor prior to obtaining a baseline blood sample. All enrolled patients underwent PPCI with 27 patients receiving ticagrelor alone (T cohort) and 25 patients receiving a clopidogrel bolus followed by a bolus of ticagrelor prior to PCI (C+T cohort). As expected, patients in the C+T cohort were more likely to present to a referral hospital (92.0% vs. 7.4%, p<0.01) as opposed to direct transfer to the PCI center by emergency medical services. The median time from clopidogrel bolus to baseline blood sample draw was 38.1±11.7 minutes. During the study, one patient in the T cohort experienced acute ST 2 hours after the ticagrelor bolus (PRU of 240 at time of ST) and was returned to the catheterization laboratory for repeat PCI with use of a glycoprotein IIb/IIIa inhibitor. During the period of glycoprotein IIb/IIIa administration PRU values were not available due to interference with the VerifyNow assay.

The groups were well balanced in terms of baseline characteristics, including age, sex, body mass index, renal function, atherosclerotic risk factors, and cardiovascular history (**Table 1**). Similarly baseline and follow-up laboratory values were similar (**Table 2**). One patient presented in cardiogenic shock in the T cohort with the remaining patients presenting as Killip class I/II. No differences were observed in baseline blood pressure measurements though the T cohort had a higher mean baseline heart rate (83.8 vs. 71.0 bpm, p<0.05). The groups were also well balanced with regards to procedural characteristics with no differences in the infarct related artery, number of stents deployed, or complementary medical therapy administered at baseline. However, patients in the C+T cohort had longer door-to-balloon times (103 (IQR 85–126) vs. 56 (IQR 46–75) minutes, p<0.001) and onset of symptoms to balloon times (175 (IQR 161–196) vs. 120 (IQR 98–161) minutes, p = 0.009).

**Table 1:** Baseline characteristics.

	Ticagrelor (n = 27)	Clopidogrel+Ticagrelor (n = 25)	p-value
Age – years (SD)	62.3 (10.3)	57.6 (10.7)	0.11
Males – no. (%)	22 (81.5)	24 (96.0)	0.10
Hypertension – no. (%)	11 (40.7)	10 (40.0)	0.96
Diabetes – no. (%)	3 (11.1)	5 (20.0)	0.37
Dyslipidemia – no. (%)	9 (33.3)	5 (20.0)	0.28
Smoking – no. (%)	10 (37.0)	12 (48.0)	0.42
Cardiovascular history – no. (%)			
Myocardial infarction	3 (11.1)	2 (8.0)	0.70
CABG	0 (0.0)	0 (0.0)	1.00
PCI	1 (3.7)	1 (4.0)	0.96
Stroke	1 (3.7)	0 (0.0)	0.97
Killip class – no. (%)			
I/II	26 (96.3)	25 (100.0)	
III/IV	1 (3.7)	0 (0.0)	
Systolic BP – mmHg (SD)	139.1 (23.8)	146.5 (32.5)	0.35
Diastolic BP – mmHG (SD)	86.3 (16.9)	89.0 (21.9)	0.62
Heart rate – beats per minute (SD)	83.8 (28.4)	71.0 (13.8)	0.05
Body mass index – kg/m^2^ (SD)	28.6 (4.4)	28.1 (5.2)	0.72
GFR (MDRD) – mL/min/1.73m^2^ (SD)	103.2 (32)	87.3 (22.3)	0.06
Infarct artery – no. (%)			0.61
Left anterior descending	9 (33.3)	11 (44.0)	
Circumflex	2 (7.4)	3 (12.0)	
Right coronary artery	15 (55.6)	11 (44.0)	
PCI characteristics			
Door to balloon – minutes (IQR)	56 (46–75)	103 (85–126)	**<0.001**
Onset to balloon – minutes (IQR)	120 (98–161)	175 (161–196)	**0.009**
CK peak – U/L (SD)	2060.4 (1527.7)	1342.7 (1019.2)	0.054
Stent number – no. (SD)	1.3 (0.8)	1.4 (0.7)	0.60
TIMI flow			
Pre-PCI – no. (%)			0.19
0	15 (55.6)	17 (68.0)	
1	4 (14.8)	0 (0.0)	
2	3 (11.1)	2 (8.0)	
3	4 (14.8)	6 (24.0)	
Post-PCI – no. (%)			0.33
0	0 (0.0)	0 (0.0)	
1	0 (0.0)	0 (0.0)	
2	1 (3.7)	0 (0.0)	
3	26 (96.3)	25 (100.0)	
Morphine – no. (%)	3 (11.1)	5 (20.0)	0.37

BP: blood pressure; CABG: coronary artery bypass grafting; GFR: glomerular filtration rate; MDRD: modification of diet in renal disease; SD: standard deviation; CK: creatinine kinase; TIMI: thrombolysis in myocardial infarction; IQR: interquartile range.

**Table 2:** Baseline and 48 hour laboratory values.

	Ticagrelor (n = 27)	Clopidogrel+Ticagrelor (n = 25)	p-value
Baseline			
Hemoglobin, g/L – mean (SD)	145.4 (13.0)	142.0 (12.6)	0.35
Platelets, 10^9^/L – mean (SD)	248.4 (57.0)	226.0 (50.0)	0.14
Mean platelet volume, fL – mean (SD)	10.4 (1.2)	10.8 (0.9)	0.16
48 hour			
Hemoglobin, g/L – mean (SD)	133.0 (12.2)	137.5 (12.9)	0.21
Platelets, 10^9^/L – mean (SD)	205.1 (56.6)	211.8 (48.6)	0.65
Mean platelet volume, fL – mean (SD)	10.5 (0.8)	10.7 (0.7)	0.52

### Effect of Ticagrelor and Clopidogrel Plus Ticagrelor on Platelet Reactivity

**Figure 2:**
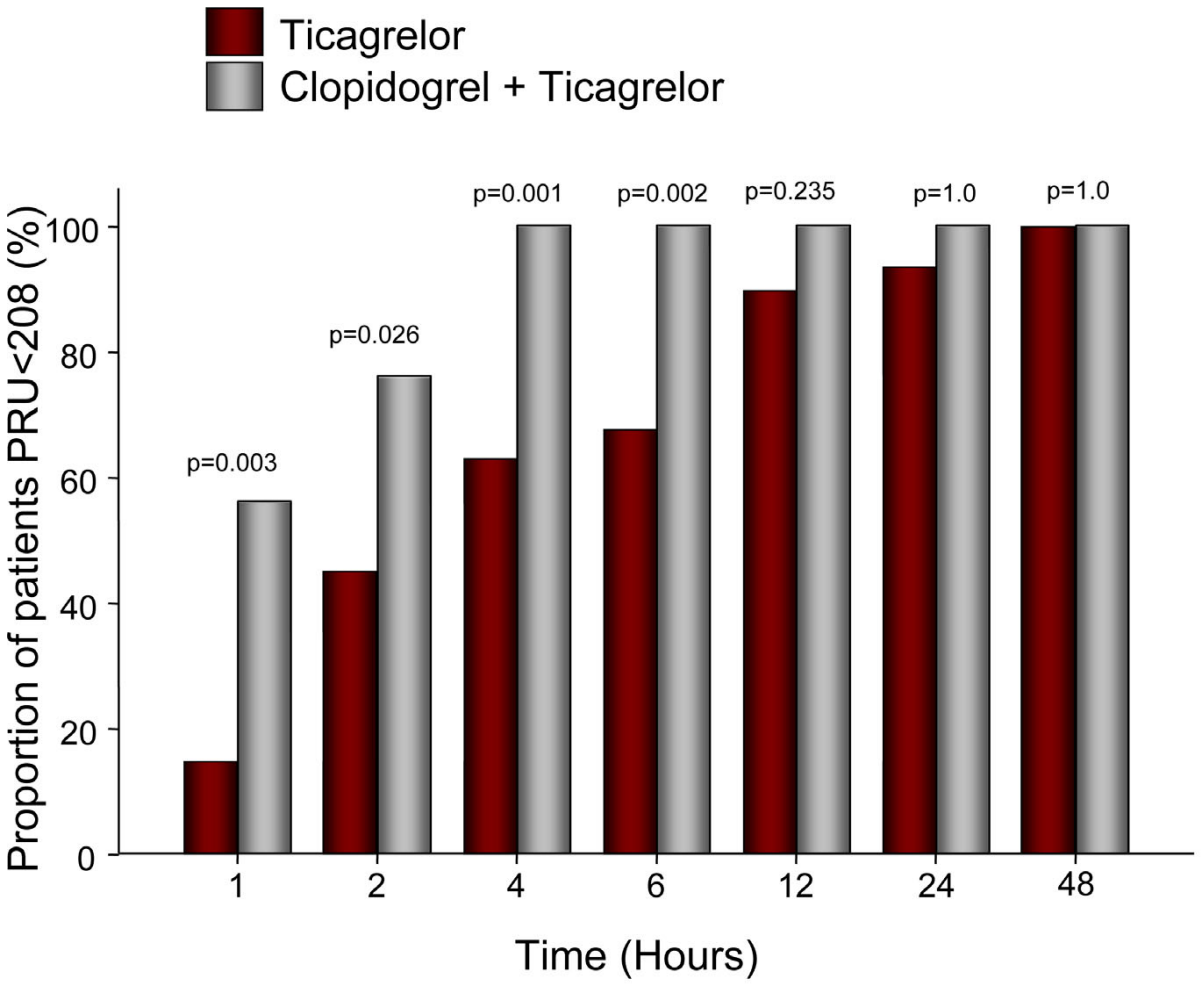
Proportion of patients achieving platelet reactivity unit values <208 during the first 48 hours following ticagrelor bolus alone (red bars) or clopidogrel followed by reloading with a ticagrelor bolus (gray bars). Clopidogrel and ticagrelor co-therapy resulted in more patients achieving target platelet inhibition at 1, 2, 4, and 6 hours following ticagrelor administration.

The primary outcome, the proportion of patients achieving target PRU<208, was significantly higher in the C+T cohort compared to T alone (76.0 vs 44.4%, p = 0.026, **Figure 2**). Of note, this difference was significant at all time points up to and including 6 hours – specifically, at 1 hour (56.0% vs. 14.8%, p<0.01), 4 hours (100.0% vs. 61.5%, p<0.01), and 6 hours (100.0% vs. 64%, p<0.01) – resulting in fewer patients with HPR in the C+T group. Furthermore, while all patients in the C+T cohort achieved target PRU by 4 hours, 3 patients in the T cohort had HPR at 12 hours with one patient in this group not reaching target PRU until 48 hours. The difference in the proportion of patients reaching target PRU was not significant at these time later time points, however.

When analyzed as a continuous variable, C+T therapy was associated with a marked reduction in absolute PRU values. As expected, baseline PRUs and hematologic laboratory values were similar between the groups (**Table 2** **&** **Table 3**). Importantly, clopidogrel administration occured an average of 38.1 minutes prior to baseline PRU assessment and its bioavailability is known to be delayed in STEMI patients [18]. However, by 2 hours, PRUs in the C+T cohort were significantly reduced compared to T alone (90 (IQR 5–205) vs. 220 (IQR 83–269), p = 0.02, **Table 3**). The difference in absolute PRU values persisted at all subsequent time points assessed.

**Table 3:** Platelet reactivity units following ticagrelor administration.

	Ticagrelor	Clopidogrel+Ticagrelor	p-value
Baseline	252 (233–280)	255 (223–304)	0.934
1 hour	249 (217–302)	203 (179–266)	0.084
2 hours	220 (83–269)	90 (5–205)	**0.019**
4 hours	103 (42–232)	14 (5–68)	**<0.001**
6 hours	52 (16.5–223)	5 (2–13)	**<0.001**
12 hours	34 (5–65)	9 (5–26)	**0.040**
24 hours	31 (9–62)	5 (3–14)	**0.002**
48 hours	37 (10–53)	5 (1–20)	**0.023**

All values represent median and interquartile range.

## Discussion

Our report is the first to study the effect of bolusing ticagrelor after clopidogrel administration on the pharmacodynamics of platelet inhibition in patients undergoing PPCI. Compared to ticagrelor alone, we observed that concurrent clopidogrel and ticagrelor was associated with a reduced risk of HPR with a greater number of patients achieving target PRUs at all time points analyzed within the first 6 hours. Moreover, the administration of a clopidogrel loading dose prior to ticagrelor resulted in a significant reduction in the absolute PRUs persisting to 48 hours, suggesting a positive pharmacodynamic interaction between the drugs. Finally, though not directly compared in our study, our findings suggest that the onset of ticagrelor action is significantly delayed in patients with STEMI compared to healthy controls – a finding consistent with a recent report comparing ticagrelor and prasugrel in PPCI [8]. Though the mechanism of the interaction between clopidogrel and ticagrelor is unknown, the early benefits of combination therapy on platelet reactivity and the associated potential clinical benefits in STEMI warrant further study.

The most important finding in our study is the pharmacodynamic impact of clopidogrel administration prior to ticagrelor – an interaction previously suggested in other studies describing cross-over from clopidogrel to ticagrelor therapy. In the RESPOND study, clopidogrel-responsive patients crossing over from clopidogrel to ticagrelor demonstrated an additional 20% platelet inhibition compared to initiation of ticagrelor alone [10]. Similarly, in a substudy of the DISPERSE-2 trial, greater platelet inhibition was observed in patients pre-treated with clopidogrel as opposed to clopidogrel-naïve patients [17]. More recently, data from a canine model similarly suggested that a pharmacodynamic interaction exists between clopidogrel and ticagrelor, but not cangrelor and ticagrelor [19]. Collectively, though suggestive of a potential positive pharmacodynamic interaction, none of the above studies assessed a sufficient number of time points to adequately profile this postulated effect. Results from our study confirm this interaction with patients in the clopidogrel plus ticagrelor cohort achieving more rapid and profound platelet inhibition than ticagrelor alone.

While recent studies have drawn into question the measurement of platelet function as a therapeutic target, there is considerable evidence to suggest that HPR is associated with adverse events in patients undergoing PCI [6], [20]–[22]. Moreover, the P2Y12 assay continues to be used as an endpoint to compare thienopyridine drugs in multiples studies [23]–[25]. Indeed, more rapid and profound platelet inhibition may be of even greater benefit in the pro-thrombotic milieu of STEMI. However, a recent report by Alexopoulos and colleagues [8] highlighted delayed pharmacodynamics of both ticagrelor *and* prasugrel in patients presenting with STEMI, with 12 of 27 patients exhibiting HPR at 2 hours. Our results replicate these findings, with 12 of 27 clopidogrel-naïve patients exhibiting HPR. In contrast, 76% of patients pre-treated with clopidogrel achieved a PRU<208 by 2 hours with no HPR observed in any patient by 4 hours. Indeed, the pharmacodynamic profile achieved with clopidogrel plus ticagrelor combination therapy more closely approximated that seen in stable coronary artery disease patients in the RESPOND and ONSET/OFFSET studies [26], [27]. This is particularly notable as in the PLATO-STEMI subgroup a trend towards benefit is seen with increasing doses of pre-randomization clopidogrel, highlighting the potential benefit of combination therapy [2]. While our study was not powered to demonstrate a link between more rapid platelet inhibition with combination therapy and reduced clinical events, it is attractive to hypothesize that the delayed onset of platelet inhibition seen in STEMI patients may result in increased peri-PCI ischemic events. Accordingly, strategies to achieve more rapid P2Y12 blockade, such as clopidogrel co-therapy with ticagrelor or pre-hospital administration of ticagrelor (as is in the ATLANTIC study, NCT01247580), are the focus of ongoing research.

Our study is certainly not without limitations, the most important of which is its non-randomized, observational design, which resulted in an imbalance in the number of inter-hospital transfers between the groups. However, the cohorts were otherwise similar with respect to baseline variables thereby rendering the large differences observed in PRUs unlikely to be the result of confounding variables. Secondly, we set out to assess the potential impact of co-therapy on the pharmacodynamic profile of ticagrelor in patients with STEMI. Accordingly, the current study was underpowered to assess the impact of more rapid platelet inhibition on clinical outcomes such as ST, re-infarction, and death. We instead elected to use a clinically validated PRU cut-off, which has previously been demonstrated to be associated with adverse clinical events, to define ‘therapeutic platelet inhibition’. The VerifyNow P2Y12 assay was selected as a modality for measuring platelet function as it has been shown to be superior to other assays in predicting clinical events [28], [29]. Finally, we did not collect samples for analysis of clopidogrel metabolites or serum levels of ticagrelor and cannot comment on the effects on the pharmacokinetic profiles of either drug. Thus, we are unable to draw conclusions regarding the potential mechanism of either the delayed onset of action in STEMI or the interaction observed in the C+T cohort. While delays in absorption in the context of STEMI have been described for clopidogrel, it is unlikely that combination therapy affects ticagrelor absorption. Rather, the pharmacodynamic interaction may reflect modulation at the level of the P2Y12 receptor – a mechanism which remains to be confirmed [18].

## Conclusions

In patients referred for PPCI, reloading with ticagrelor following clopidogrel resulted in more rapid and profound platelet inhibition, demonstrating a positive pharmacodynamic interaction and resulting in fewer patients with HPR at 2 hours. This interaction has important implications for both clinical and pharmacodynamic studies. The impact of this interaction on cardiovascular events remains to be established.
